# Understanding the experiences of ketogenic metabolic therapy for people living with varying levels of depressive symptoms: a thematic analysis

**DOI:** 10.3389/fnut.2024.1397546

**Published:** 2024-06-06

**Authors:** Erin L. Bellamy, Florentia Hadjiefthyvoulou, James Walsh, Jennie Brown, John Turner

**Affiliations:** ^1^School of Psychology, University of East London, London, United Kingdom; ^2^School of Health Sciences, City, University of London, London, United Kingdom

**Keywords:** depression, thematic analysis, diet adherence, human, ketogenic diet, qualitative research, quality of life

## Abstract

**Background:**

Evidence suggests that a ketogenic diet (KD) may help to alleviate psychiatric symptoms, including depression and anxiety. Positive changes have been reported such as improvements in cognition, concentration, and sleep, a reduction in hunger, and an increase in well-being, energy, confidence, and resilience. This research aims to understand the impact of a non-calorie-restricted KD on depression and aspects of psychological well-being in those with varying degrees of depressive symptoms. Though there are a few studies directly exploring the experiences of those following a KD, this will be the first study to explore the narrative from a mental health and psychological well-being viewpoint.

**Method:**

A sample of nine participants who had followed a non-calorie restricted KD intervention of 50 g of carbohydrates or less per day for at least 12 weeks were recruited. Participants were split into ‘healthy adults’ group who had no to low depressive symptoms and ‘depressive symptoms’ group who had mild to moderate depressive symptoms. A reflexive thematic analysis was considered suitable for this study.

**Findings:**

Five core themes and 24 subthemes were created. These were, (1) Poor health prior to program; (2) Hunger and cravings-the food and mood connection; (3) Psychological well-being improvements; (4) It becomes a lifestyle; and (5) Implementation difficulties. Participants experienced mental health improvements such as increased self-esteem, confidence, motivation, and achievement. Some experienced more control in life and a greater sense of reward. Those with depressive symptoms who initially reported low self-worth and hopelessness later reported increased self-esteem and renewed meaning and purpose in life. The findings from this study reflect the previous reports that the diet implementation can be difficult initially, but soon becomes easy to follow and turns into a lifestyle.

**Conclusion:**

In the literature, there are very few qualitative studies that explore the accounts and lived experiences of those following a KD. From the participants’ accounts in this study, it appears that the benefits and positive outcomes of this diet outweigh any negative side-effects experienced. This is encouraging for those who are looking for adjunctive therapies to address and improve their depressive symptoms and overall mental health.

## 1 Introduction

The ketogenic diet puts the body into ketosis, a metabolic state which utilizes fat and ketones as a primary fuel source (1). Evidence suggests that the KD produces significant metabolic changes in the body that influence mood and depressive symptoms (2–4). According to previous research, the presence of ketones in the body has been shown to be neuroprotective, promote mood stabilization, and may improve symptoms of depression (5–7). The theoretical basis for this is that the ketones provide an alternative energy source to the brain which can regulate many biological processes that have become dysregulated due to biological or psychological factors (8–11).

To date, many of the studies on the KD and psychiatric conditions have focused on mechanisms of action in rodents and mice, and less so in humans (12–15). Though human studies have recently been published they are predominantly case studies or initial pilot studies (6, 16–19) no randomized control trials have been carried out to investigate the impact of the ketogenic diet on psychological well-being or depressive symptoms, though they are now underway.

Studies looking at the effects of the ketogenic diet on psychiatric conditions have been published as far back as the 1960’s (20). Research into the ketogenic diet’s effects on bipolar depression type 1 (21) and bipolar depression type 2 (22) in the past decade has suggested that the diet has mood stabilizing effects and may reduce the need for psychiatric medications. More recently, a study using the ketogenic diet for serious mental illness in an inpatient hospital setting of 28 patients, found 43% clinical remission and 64% of patients discharged on less medication than when they started (16). Randomized controlled trials (RCTs) are in progress to investigate whether a ketogenic diet can be used as a therapeutic medical intervention for psychiatric illness. However, the research available at present is predominantly quantitative in nature.

It is important to carry out qualitative research in these populations to better understand the experiences, both positive and negative, of those following specific diets. A recent publication in the area of ketogenic metabolic therapy for mental health has called for more qualitative research to be carried out in this area. The authors state that the changes clinicians see in their patients may not always be quantifiable and therefore without a qualitative perspective, there is a risk that not all positive and negative changes are being adequately captured (23).

When reviewing the low carbohydrate and ketogenic diet literature, it is only within the last 5 years that qualitative studies on the ketogenic diet have been published. This may be a result of the ketogenic diet gaining popularity in recent years as a dietary approach to lose weight. Harvey et al. (24) looked at healthy adults, non-obese, non-diabetic, following a ketogenic diet with medium chain triglycerides (MCT) oil supplementation (*N* = 28), specifically the ‘lived experience’ of such individuals over 3 weeks. Participants experienced benefits in well-being, mood, and sugar cravings and these improved gradually over the duration of the study. They found that mood, energy, and cognition were low at the start of the study, most likely due to an immediate reduction in blood glucose with a slower increase in ketones, as the participants had not yet reached a state of ketosis. This was also true for satiety levels, hunger, and the desire to eat, as well as sugar cravings, all of which improved as time went on and participants achieved a state of ketosis, where ketone levels increased and provided energy for the body. Individuals who came off the diet or who were non-compliant with it, experienced negative effects in the form of a “food hangover.”

Sleep quality also improved which is to be expected as research suggests that following a higher carbohydrate diet negatively impacts sleep by increasing sleep length but reducing its quality by spending more time in rapid-eye-movement (REM) compared to slow-wave sleep (SWS) (25). According to a systematic review, individuals following a low carbohydrate diet tend to spend more time in SWS and experience an increase in the duration of deep sleep (26).

Using thematic analysis, Newson and Parody (27) looked at individuals’ experience of low carbohydrate diets (LCD) in those living with T2D. They found that in 10 participants who had been following a LCD for at least 5 months, they experienced a lack of hunger, gained confidence, felt resilient, calm and more energetic. Although they felt starting the LCD was difficult, it was easier over time, and soon became a lifestyle.

Wong et al. (28) looked at those with type 1 diabetes and T2D who followed a ketogenic diet for between six and 19 months (*N* = 14). Using thematic analysis, they found that individuals experienced greater glycemic control, weight loss and satiety. Participants also experienced improvements in cognition, specifically concentration, a reduction in chronic pain levels, an increase in well-being and energy, and improvements in sleep. Individuals reported no hunger and stated that the KD was easier to follow than other diets, however they did initially express difficulty getting used to the idea of eating a KD as the foods eaten are not in keeping with conventional nutritional guidelines. Individuals reported some keto flu or keto adaptation symptoms at the start of the diet such as fatigue, headaches, dizziness, and constipation but these symptoms were temporary and soon passed. These findings support the work of Bostock et al. who found that keto flu symptoms were apparent when starting the diet but that they were transient (2020).

According to Wong et al. (28), Newson and Parody (27), and Bostock et al. (29) the diet implementation can be difficult initially, but soon becomes easier to follow and progresses into a lifestyle. The findings from the current study reflect these previous reports. From this literature, it is clear that the ketogenic diet can lead to improvements in mental health and relief from psychiatric symptoms in some people. The benefits, if a patient responds to the diet, can be life changing, and the safety of the diet has been confirmed (30–33), but whether the diet is easily implemented and sustainable in the real-world within the general population is uncertain. Research is required to investigate the accounts, perspectives, and experiences of those following a ketogenic diet to better understand their journey to improve their physical and mental health. This will help researchers to better inform care pathways and provide the right support to individuals at the right time.

Research suggests that some individuals following a ketogenic diet may experience improvements in their psychological well-being, though it is unclear which population would benefit most from this dietary intervention and what ‘barriers to entry’ they might face when initiating the diet (5, 18, 34).

The aim of this current study is to review the accounts of participants who have completed an online ketogenic dietary program through a remote care model and to identify any common themes relating to their journey. This study focused specifically on the health of participants prior to the start of the program, the challenges and obstacles they faced implementing the diet and any physical or psychological changes, either positive or negative, that they experienced throughout the program. Aside from directly discussing their accounts of the diet, the interviews also covered participants’ overall health and well-being in a broader sense and touched on areas such as their relationship with food, their general health, and their mental and physical state prior to starting the ketogenic diet.

Through reflexive thematic analysis, the accounts and attitudes of participants following the ketogenic dietary intervention were explored. Though there are a few studies directly exploring the experiences of those following a LCD or ketogenic diet, this is the first study to explore the narrative through the lens of mental health and psychological well-being. The findings will inform future research directions into the application of a ketogenic diet and ketogenic metabolic therapy either through online programs or via healthcare professionals in clinical practice. The data gathered will also help to understand the utility of ketogenic metabolic therapy more fully for those with depressive symptoms and poor psychological well-being.

## 2 Materials and methods

### 2.1 Design

The study was nested within a randomized controlled trial (RCT) on the impact of the ketogenic diet on depression and psychological well-being. This was an interview-based research piece, carried out by the first author, using face to face semi-structured interviews of participants drawn from the RCT sample of ketogenic dieters and using Braun and Clarke’s reflexive thematic analysis process to draw out, create, and analyze themes (35). Reflexive thematic analysis takes an experiential approach and was chosen to understand the views, perspectives, and perceptions of participants following the ketogenic dietary intervention (36).

### 2.2 Participants

#### 2.2.1 Collaborators—Diabetes Digital Media Ltd.

Since 2007, Diabetes Digital Media Ltd. (DDM) has been providing lifestyle intervention programs and community support for those with diabetes. Their Low Carb Program currently has over 475,000 members and is the world’s largest low carbohydrate intervention. DDM provided the researcher with access to their Low Carb Program. They gave the researcher access to the program and permission to create a bespoke ketogenic diet version with aligned support materials. The program included dietary recommendations to focus on unprocessed foods with the goal of reducing carbohydrates to <50 g per day, educational videos on a variety of topics including macronutrients, food swaps and ketone testing, methods to track progress and supportive forums for peer support.

#### 2.2.2 Participants

A sample of nine participants who had followed the ketogenic diet intervention arm of the RCT were recruited for this study. The ketogenic diet consisted of no more than 50 g of net carbohydrates per day, with fat and protein *ad libitum* and a focus on whole foods was encouraged. The sample consisted of five participants who had been previously placed in the ‘healthy adults’ group for the RCT and four participants who had been placed in the ‘depressive symptoms’ group. Groups were determined based on participants scores on the Patient Health Questionnaire (PHQ-9) which measures the severity of their depressive symptoms (37). Those with little or no depressive symptoms (<5 on the PHQ-9) were included as the ‘healthy adults’ group and those with mild to moderate depressive symptoms (5–19 on the PHQ-9) were included as the ‘depressive symptoms’ group.

Out of the five in the ‘healthy adults’ group, one participant had a diagnosis of depression, and had been taking antidepressants for more than 3 weeks as defined in the RCT eligibility criteria. However, when randomized to a dietary intervention, their PHQ-9 score was three, suggesting low to no depressive symptoms and therefore they were allocated to the healthy adults group rather than the depressive symptoms group.

In the healthy adults group, there were four females and one male, and in the depressive symptoms group there were three females and one male. The mean age overall was 51 years (SD 8.12). The mean age for healthy adults was 52 years (SD 10.23) and for depressive symptoms it was 49 years (SD 5.47). All nine participants were renamed for the purpose of this analysis in order to maintain their anonymity. Reference names are seen in Table 1 along with their group allocation, age, and gender. To participate in the RCT, participants were deemed eligible based on their answers to a baseline screening questionnaire. Detailed inclusion and exclusion criteria for the RCT will be published, but an overview can be seen in Table 2.

**Table 1:** Demographics of participants, anonymized name, psych group, age, and gender.

Participant number	Anonymized name	Psych group KD	Age	Gender
1	Amari	Healthy	36	Female
2	Anika	Healthy	63	Female
3	Diane	Healthy	58	Female
4	*Harriet	Healthy	54	Female
5	Mark	Healthy	50	Male
6	Jessica	Depressive	56	Female
7	Philip	Depressive	43	Male
8	Sarah	Depressive	47	Female
9	Whitney	Depressive	50	Female

*Participant had a diagnosis of depression but showed very few depressive symptoms on the PHQ-9 and so allocated to healthy group.

**Table 2:** Criteria of inclusion and exclusion for RCT eligibility.

Criteria	Included	Excluded
Age	19–65	<19, >65
Location	UK	Outside the UK
Body Mass Index (BMI)	>18.5 kg/m^2^	<18.5 kg/m^2^
Diabetic Status	Non-Diabetic	Pre-Diabetic, T1D, T2D
Physical Health Status	No Physical Health Issues	Physical Health Issues
Pregnancy Status	Not pregnant and no plans in next 6 months	Pregnant or planning pregnancy in next 6 months
History of Recent Weight Loss	Less than 12.7 kg	12.7 kg or more
History of Using a Low Carbohydrate or Ketogenic Diet in last 2 years	No History	History
Partaking in Trial Status	Not currently partaking in trial on diet or exercise	Partaking in other trial on diet or exercise
Mental Health Diagnosis Status	Depression and anxiety only	Any other mental health diagnosis
Severe Depression - High Risk Status Question ‘Recently have you had thoughts that you would be better off dead or of hurting yourself in some way?’	Answered ‘No’	Answered ‘Yes’ and referred to mental health support services
Antidepressant Medication Status	Taking Antidepressants for more than three weeks	Taking Antidepressants for less than three weeks
Patient Health Questionnaire (PHQ-9)	Scores of less than 20	Scores of 20 or greater

#### 2.2.3 Materials and measures

For the semi-structured interviews, open ended interview questions with a series of prompts were developed by the researcher in order to extract the full journey from the eligible participants. Examples of the questions included in the interview were ‘how had you been feeling psychologically and emotionally before starting this program?’ and ‘please can you describe your journey on the diet?’, with prompts such as; ‘what were reasons for joining the program?’, ‘how do you think diet played a part in how you felt?’, ‘what was your opinion of yourself?’, ‘how did you feel in the first few days?’ and ‘how has your mental state or mood changed?’

#### 2.2.4 Ethical protocol

The research protocol for this qualitative investigation was approved by the University of East London, UREC 1718 87 on the 4^th^ of July 2018 and interviews were carried out between April and June 2019. Participants provided their written informed consent to participate in this study and were reminded that they could withdraw from the study at any point. Participants consented to the recording of interviews, which were anonymized and transcribed. Interviews were stored on an encrypted computer, which housed all data.

#### 2.2.5 Procedure

Participants must have completed 12 weeks of the trial to be eligible for this study as it was imperative that they could give a full account of the ketogenic intervention from start to finish. Thirty-three participants were identified as eligible for the study. Participants were contacted and asked if they would take part in the follow up interview. Once participants had been recruited, read, and understood the information sheet and signed the consent form, the researcher agreed a time and date to carry out the interview. Interviews were approximately 30 min long, with some a little longer based on how much the participant wanted to share. The researcher reached data saturation with nine participants. This is the point where no additional themes or insights would emerge from continued data collection.

Participants were encouraged to share their journey of following the online dietary intervention and the positive and negative effects they encountered during their dietary change. Following the interviews, all participants were debriefed and thanked for their participation in the study. Interviews were transcribed and full transcripts were then uploaded to data analysis software NVivo to assist with the qualitative analysis. Once uploaded, the audio recordings were subsequently deleted.

#### 2.2.6 Epistemological position

An essentialist and realist approach to the data was taken, with the language used by participants to share their accounts of the intervention taken at face value, with no further in-depth interpretation of the meaning of language used seen as necessary. What participants say is their experience and reality (38). Then, an inductive or ‘data-driven’ approach was taken as there were no specific research questions and no pre-defined themes for this study. This was an exploratory analysis where the themes became analytical outputs and were reflective of the data collected and were therefore free from the researcher’s analytical preconceptions (39). Themes were actively identified at the semantic level, meaning that no further interpretation of the data was carried out beyond what the participants had shared. Themes were then described and further interpreted. This position and approach are similar to that used by Newson and Parody (27) who used thematic analysis to investigate the experiences of those with T2D following a LCD. Discussions occurred between authors throughout the study process to ensure a shared understanding and agreement on the final themes.

#### 2.2.7 Analysis

**Figure 1:**
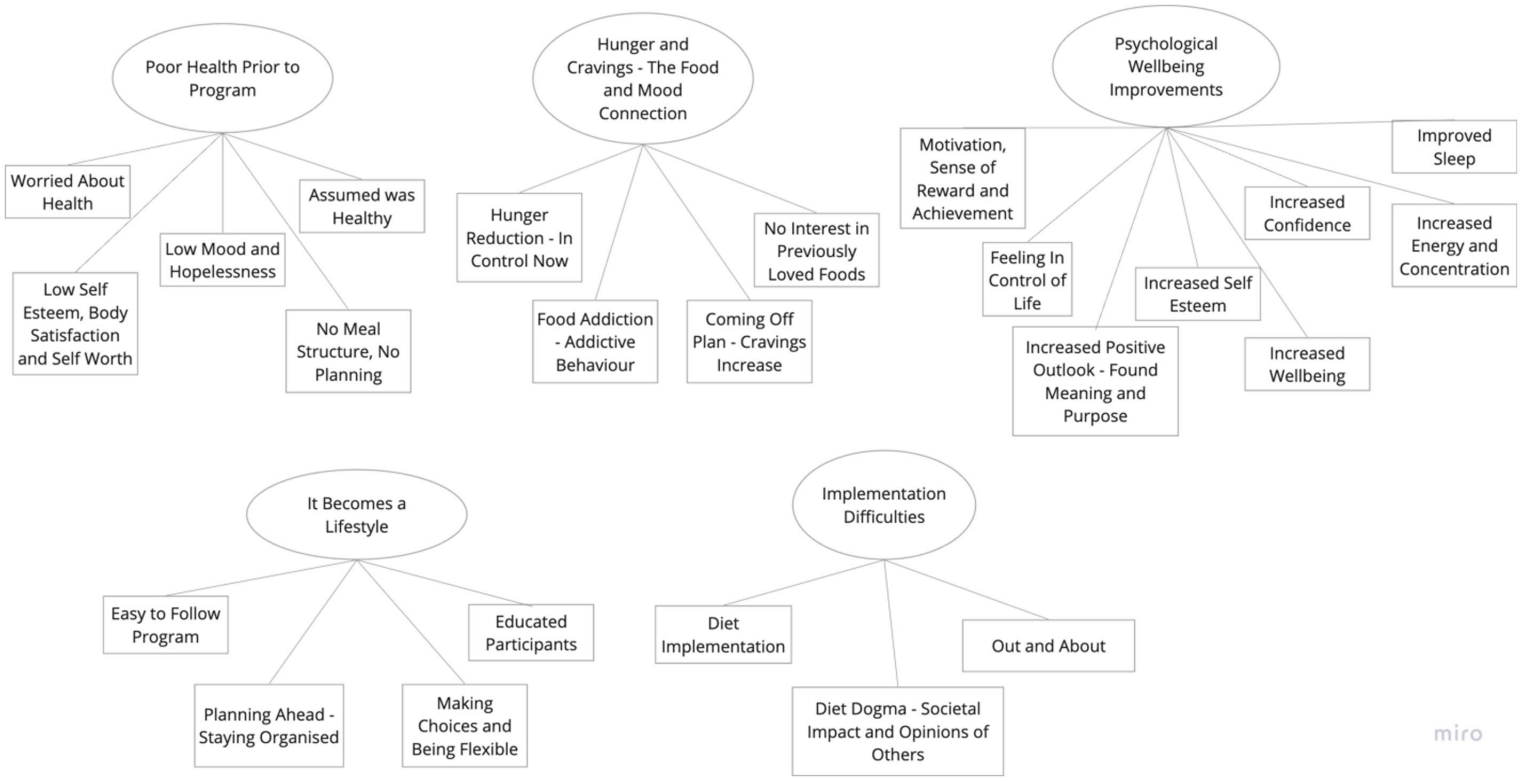
Thematic map showing five main themes.

An inductive thematic analysis of the interview transcripts was carried out following the six-phase framework set out by Braun and Clarke (38), to review the shared accounts of participants. Transcripts were initially coded line-by-line and sorted. The researcher became familiar with the data by reading and re-reading the transcripts before generating initial codes. These preliminary codes were assigned to the data to describe the content. The researcher then searched for consistent patterns and developed themes from the codes across all interviews after a period of familiarization with the collected data which is in keeping with the work of Terry et al. (40). Once themes were constructed, they were repeatedly reviewed, defined, and renamed. There were five main evident themes with three to eight sub-themes each, see the final Figure 1. The findings were kept to five themes so that the analysis stayed coherent, and that the researcher could provide a meaningful overview of the data. This is in keeping with the guidance from Braun and Clarke (35).

## 3 Results and findings

### 3.1 Findings in relation to groups and previous research

Five core themes and 24 subthemes were created during this analysis as seen above. The five core themes in order from highest referenced to lowest referenced were, (1) Poor health prior to program; (2) Hunger and cravings—the food and mood connection; (3) Psychological well-being improvements; (4) It becomes a lifestyle; and (5) Implementation difficulties. Direct quotes from participants have been included to help illustrate each theme and subtheme and, in some cases, the group they were allocated to has been identified to better understand if a subtheme was predominantly representative of one specific group rather than another.

### 3.2 Theme 1—Poor health prior to program

This theme is characterized by participants stating that they felt generally healthy, however as the theme was refined, physical and psychological health issues arose from the data which suggested that people were not as healthy as they had initially assumed.

#### 3.2.1 Subtheme—Assumed was healthy

The first question in the interview asked how the participants health was before starting the program. Four participants out of nine, “assumed they were healthy” and that they ate in a generally “healthy” way. Diane believed that her health was “pretty good in general,” both now and before they started the program. Whitney stated that she could not remember the last time she purchased a ready-made meal as she always prepares her meals at home alluding to the fact that she followed a healthy diet overall. Mark shared his thoughts when grocery shopping:

*“You know, I’d go round the supermarket and look at people’s trollies and think ooh no you do not want to be eating all that crap because I thought we were, well we were, we do, eat relatively sensibly.”* (Mark).

Some participants were following a standard western diet prior to starting the ketogenic diet. Other diets such as the Mediterranean diet, which is lower in processed foods, are associated with greater psychological well-being compared to the western diet (41). Perhaps some participants experienced this when moving from the standard western diet to the ketogenic diet.

#### 3.2.2 Subtheme—No meal structure, no planning

Though answers to the interview questions about health varied from good to bad, the overarching observation was of poorer health prior to initiating the program. There appeared to be a lack of meal structure and little planning of food or meals in advance which led participants to graze through the day, eat on the go, or eat less healthy options based on what was available when they were faced with hunger. This was reported by two participants in the depressive symptoms group and one in the healthy group. The lack of meal structure and food planning was felt by Sarah who shared that because she only had to cook for herself, she did not, and for Harriet, she would spend the day “eating rubbish” and skipping dinner as a result. For Whitney, a morning routine with no meals planned appeared to be normal:

*“So, then I would not have time for breakfast so would just go to work and drink coffee…There is that, if there’s biscuits going round, or cake, you would have that before your lunch.”* (Whitney).

Participants appeared to have a daily self-care routine that lacked a meal structure or food planning stage. The perceived lack of time to plan meals or prepare food is associated, unsurprisingly, with a higher intake of fast and convenient food (42). On the other hand, meal planning has been linked with improved diet quality and less obesity (43) and structured meals may increase the success of weight loss (44) and therefore overall health if employed consistently.

#### 3.2.3 Subtheme—Worried about health

There appeared to be an underlying feeling prior to the program, of worries about current and future health, understandably. Again, this questions how participants truly felt about their overall health prior to the program. Though just under half of the participants stated they felt generally healthy, once the researcher dug a little deeper there appeared to be ongoing health concerns among many. Philip stated that though he had not been morbidly obese, he had always been on the “wrong side of ok” and that was a concern for him. The idea that some participants needed to take medications for their ailments was also a cause for worry. Amari stated that she did not want to take the medication and it “does not make me feel nice.” When participants were asked to share more about their experience of health prior to the program, it became clear that there were both significant negative physical and psychological symptoms present in their everyday lives. Physically, participants experienced extreme tiredness, fatigue, and lethargy throughout the day.

*“I was really struggling in meetings at work, and I was constantly falling asleep, and I kept thinking, what on earth is wrong with me!”* (Harriet).

*“Tired is an accurate word of how I would describe myself, sluggish and just tired. I did not have a lot of energy anymore, restless, you know.”* (Sarah).

Prior to the dietary intervention their cognition and ability to concentrate was also impaired, they lacked focus and often experienced symptoms such as brain fog. Mark stated that he had always had a low attention span, and that he still struggles with that now. Philip shared his more debilitating experience:

*“Whether it’s a funk or fog I do not know, so this is where I sort of started, I could not really sort of keep any concentration and any focus or goal…I’ve always enjoyed learning, but I could not focus, I could not retain any attention to what I was doing…I was getting frustrated with myself.”* (Philip).

#### 3.2.4 Subtheme—Low self-esteem, body satisfaction and self-worth

Psychologically, extremely low self-esteem, poor body satisfaction and little self-worth were evident in four of the nine participants. Worthlessness is a symptom identified in the diagnosis of depression and research shows that poor body satisfaction predicts worse depression and mood outcomes (45, 46). Three participants from the depressive symptoms group stated that they felt “terrible about myself” (Jessica), that they were “feeling so rotten” (Whitney), and “my opinion of myself has never been terrific” (Philip). One participant from the healthy group shared that:

*“I could not even look at myself in the mirror, really, really, low self-esteem.”* (Amari).

This was followed by “rock bottom” confidence (Jessica), a lack of motivation, and the feeling they were “stuck in a rut.” Understandably, day to day energy was limited and carrying out simple daily routines and tasks was a struggle for some participants. As Jessica put it, “I could not motivate myself to do anything.” Whitney shared her thoughts:

*“Yeah, so I suppose a lack of motivation, you just get into that rut do not you, you feel lazy so you just, you cannot get up…and just felt stuck in a rut.”* (Whitney).

This lack of motivation, and low energy that participants experienced ties back to their difficulty of following a routine that supports a healthy lifestyle and may have been a contributing factor to their lack of daily structure with regards to meal timing and food planning. Whitney again shared an example of this:

*“Not getting out of bed on time to get myself ready for work to get to work…Yeah just feeling pretty lazy actually.”* (Whitney).

#### 3.2.5 Subtheme—Low mood and hopelessness

For some participants, their mood was extremely low which is a symptom necessary for the diagnosis of depression (46). From the healthy group, Anika felt “quite depressed really,” and from the depressive symptoms group, Philip experienced a sense of hopelessness.

*“I was beginning to worry about, not depression itself but being in a rut if you know what I mean…I felt pretty hopeless, I wasn’t suicidal, I just could not work out what was wrong with me.”* (Philip).

Jessica even felt a lost sense of meaning and purpose in their life.

*“I was just getting really down about, well where is my life going and what was there left for me to do…but I could not find my niche in life…you lose your identity really…you just think, oh well, who am I then? Where do I fit in?”* (Jessica).

These low feelings and experiences were to be expected in some participants as they showed mild to moderate depressive symptoms at the start of the study which is why they were placed in the depressive symptoms group. However, their accounts of daily life prior to the program are clearly impactful. What is interesting is that there is a positive relationship between the western diet, and major and persistent depression (47) which could in part explain their perceived low mood. The western diet is also associated with metabolic syndrome which is associated with depression (48–50). Overall, these findings suggest that poor physical and mental health was experienced by some participants prior to starting the ketogenic diet regardless of how individuals scored on the depression scale (PHQ-9).

### 3.3 Theme 2—Hunger and cravings—the food and mood connection

The second theme from this data is Hunger and Cravings – The Food and Mood Connection. This theme starts by explaining how participants experienced hunger and cravings during this program, followed by their experience of an increase in self-awareness where they were able to make the connection between eating certain foods and the impact thereof both physically and mentally.

#### 3.3.1 Subtheme—food addiction—addictive behavior

Many participants noted that consuming sugar through sweets, chocolates, or baked goods, had a negative impact on their physical and psychological health. They found that when they ate sugar or carbohydrates, they “crave it more” (Whitney). Harriet shared how her cravings could impact her behavior around sugar:

*“If I do not have any sugar, I do not crave it but the minute I have some, then I just go off the scale again.”* (Harriet).

When she attempted to eliminate the sugar from her diet, she experienced emotional withdrawal like symptoms. This may sound extreme, but sugar addiction (51) has been shown to be equally or more addictive than other substances such as cocaine (52–54) and food addiction has been recently considered a valid diagnostic construct (55). Research suggests that the western diet can promote addictive eating behaviors due to the composition of many ultra-processed convenience foods which are high in fat, salt and sugar, the combination of which is not found in natural whole foods (49). Interestingly a recent case series shows promise for treating Binge Eating Disorder and food addiction symptoms with a ketogenic diet (56). A pilot study by Rostanzo et al. (57) of five participants using the KD as a treatment for binge eating and food addiction in women found that after following a KD no cases of food addiction or binge eating were recorded, and all participants improved.

In the current data, there were three accounts from participants on their experience of sugar withdrawal.

*“I have to go through that real craving few days where I feel like I need to be locked in a room so I cannot have any, and then I’ll be fine.”* (Harriet).

*“How your brain just thinks, it makes you believe it will be ok to have one, I can account for this it will be ok, but no it will not be one, it never ever is, and even now, I know that, the sensible side of me knows that but the craving and desire was so strong.”* (Harriet).

*“I could demolish a packet of biscuits without even thinking about it, I could honestly, if the biscuits are in the house, I can eat them, they are in the house and they are there and they are calling me all the time.”* (Jessica).

These accounts of sugar withdrawal are similar to the behavior effects noted in other addictions. Research suggests that highly processed foods such as sugar can trigger addiction-like symptoms and behaviors, including withdrawal when restricted or reduced in some people (58, 59). These behaviors are linked to alterations in the brain’s neurochemistry, such as in the dopamine pathway, which is also altered by other addictive substances (51). In rat studies, the withdrawal symptoms from sugar were found to be similar to symptoms of morphine or nicotine withdrawal (60). Alongside this, physical symptoms of withdrawal were also experienced, and one participant could feel the impact of sugar on their blood glucose levels. Diane said:

*“Weird headachy thing when I feel my sugar go up. You know what I mean, it’s like a sugar spike kind of feeling, I feel a bit muddy headed and a bit groggy.”* (Diane).

Another participant realized that they were experiencing a change in blood glucose levels post sugar intake. They realized after they had eaten some sugar, that they were experiencing the sugar spike. They had heard about it happening before and had thought “really does that exist?” (Harriet).

#### 3.3.2 Subtheme—Hunger reduction—in control now

Once participants had started the ketogenic diet, hunger levels appeared to drop, cravings dissipated, and they felt more in control of their diet. This is in keeping with previous research which has shown that cravings for starchy foods and sweets disappear (61), appetite is suppressed, and satiety levels are heightened on a KD due to the physiological state of ketosis (62–64).

If the KD is implemented correctly, and ketosis is the goal, ketone bodies are produced and begin to rise, reaching 1-2 mmol/L after approximately 48–96 h of reduced carbohydrate intake (65). Ketones have appetite suppressing effects and therefore it is expected that hunger levels will drop (66, 67) once ketones begin to rise. Physical cravings should also reduce once blood sugar levels regulate and ketones rise (68, 69), leaving only emotional cravings like eating in response to negative emotions (70, 71) such as when anxious or bored, or even when happy (72).

Participants began to experience the drop in hunger and the reduction in frequent and bothersome food-related thoughts. Though they expected to be hungry following this diet, as they had been on previous diets, the hunger occurred only once or twice, if at all. This is in keeping with the findings from Newson and Parody (27) whose participants also acknowledged a reduction in hunger. Participants stated, “I just do not seem to get hungry at all” (Jessica) and “I’m actually eating less now than I was before” (Mark), and “I do not even think about food” (Harriet). Philip shared his thoughts:

*“It seems to be really easy, it’s like the simplest thing in the world is to not eat, rather than worry about it…I’m not a scientist, but I put that lack of hunger down to the lack of carbs.”* (Philip).

Similar to hunger, although participants were expecting to crave certain foods, little to no cravings were experienced. Some were experienced in the first few days as mentioned above, but not to the extent that was expected. Amari stated that:

*“I thought I might crave, I do not know something savoury or something sweet and I really have not. It’s been fine.”* (Amari).

Over the duration of the program, cravings appeared to reduce with ongoing low carbohydrate intake and little sugar. One participant stated succinctly, “Because I’m not having it, I’m not craving it” (Whitney). This is to be expected as physiologically, once sugar is ingested, blood sugar rises and ketones drop (73). Blood sugar and ketone levels have an inverse relationship (74). This blood sugar spike will decrease once more and leave the individual seeking more sugar and carbohydrates to increase their blood sugar levels. This is known as postprandial hunger (75). With the reduced sugar intake, individuals can avoid this cycle of sharply rising and falling blood sugar levels.

#### 3.3.3 Subtheme—no interest in previously loved foods

Three participants from the healthy group and one from the depressive symptoms group shared that as time went on, they no longer wanted or liked the taste of anything sweet, “I think my body is used to not having anything sweet in my life now” (Amari). This is in keeping with the research which shows that taste intensity can change, and that sweet receptors can become sensitized as there is no longer a frequent influx of sweet tastes (76). Harriet sums up the experience:

*“Because I have such a sweet tooth, once I stopped that I was amazed at how I could just, I had no interest in anything sweet.”* (Harriet).

Though cravings were reduced or even eliminated when following the diet, cravings could return for two reasons. Firstly, if carbohydrate filled foods were observed and looked appetizing, such as at social events, or other events where it was difficult to remove them from sight. Sarah mentioned how her workstation was surrounded by chocolate bars. Having these sugary treats around throughout the day when emotions and stresses can occur and at times overwhelm, is not ideal. Research has shown that willpower to resist sugar as a pick me up is difficult when feeling stressed or overwhelmed throughout the day (77). These foods are better off ‘out of sight and out of mind’. Mark said:

*“I think a lot of the time I’m not hungry or craving anything and then you see something, and you go hmm.”* (Mark).

#### 3.3.4 Subtheme—Coming off plan—cravings increase

Secondly, if participants came off plan or increased their carbohydrate intake enough to come out of ketosis, their blood glucose, cravings, and hunger would increase, and some participants felt like they were “back to square one” (Harriet).

*“On the Saturday, we had pizza and, on the Monday, not only did I get that horrible hunger, but I was in a tetchy mood.”* (Philip).

As Philip mentioned, not only did coming off plan increase his cravings and hunger again, but for some participants, their mood and physical health were negatively impacted as they likely experienced symptoms of keto flu as they moved back into a state of ketosis. Philip followed on with:

*“I’ve really noticed the difference to my mood. I notice now, the next day, I’m really irritable and again I’m putting it down to eating too much or eating too much of the wrong things.”* (Philip).

One participant mentioned that they experienced quite severe side effects from eating a lot of carbohydrates in one sitting at a wedding:

*“The day after, I came out in a rash, so my eye swelled up and yeah, just dreadful and that week felt just a bit rotten really.”* (Whitney).

Understandably, for most participants, their interest in previously loved foods soon decreases and they no longer miss foods they used to eat either because of how their body now responded to those foods or because their tastebuds and food preferences had changed. Wise et al. (76) reported that a reduction in sugar intake led to an increase in perceived sweetness; however, research is unclear as to whether a reduction in sugar intake changes food preferences. The draw toward foods high in sugar or carbohydrates is no longer present and participants stated that going without old favorites “does not bother me anymore” (Sarah), they have “no interest in anything sweet” (Harriet) and well, “I do not feel like it” (Whitney). Mark shared how certain he was about the dietary change:

*“I really do not miss anything and in fact the thought of eating a plate of pasta now or potatoes fills me with dread.”* (Mark).

It appears that participants have experienced a shift in their attitude toward the diet and their approach to food and the role food plays in their life. They appear to have a heightened sense of self awareness that wasn’t apparent prior to starting the program. The chaos and decision fatigue around ‘dieting’ day to day is no longer present. Mark shared that he could go out to a restaurant and “just get on with it without making a fuss” and Jessica felt that food is no longer the be all and end all of the day, “I do not think about it like I used to,” “It has been a complete revelation to me,” said Whitney. The increase in self-awareness is summed up well by Philip who said:

*“Now I can catch myself when I know I’m tetchy about nothing in particular, I’m aware of that now.”* (Philip).

Overall, participants experienced a significant reduction in their hunger and cravings which only increased when tempted or if they veered off plan with higher carbohydrates either on purpose or by accident. Participants observed and were later able to identify the negative effects that increased sugar and carbohydrate intake had on their mood and psychological well-being.

### 3.4 Theme 3—Psychological well-being improvements

This theme discusses the psychological changes and improvements that participants experienced over the course of the program. Most interestingly, what appeared to be relatively low self-reported well-being prior to the start of the program, seemed to increase over the duration of the study. This may have been due to an overall improvement in metabolic health. Evidence suggests that ketogenic metabolic therapy, and the state of ketosis, has broad positive effects across multiple metabolic pathways. This can be seen in the research, see Sethi and Ford (34) and Kraeuter et al. (78) for a review of this area.

#### 3.4.1 Subtheme—Motivation, sense of reward and achievement

An increase in motivation, determination, achievement, and a sense of reward was felt by some participants. In theme 1, “poor health prior to program,” participants stated that other diets had not worked for them and that they felt they lacked motivation daily. Since following the program, participants said that they were “determined” now (Sarah) and that “It’s nice to feel you are doing something good for yourself” (Diane). Participant Amari shared her account of the diet:

*“It’s just worked amazing well compared to anything else I’ve ever tried I feel great, I feel fantastic with it.”* (Amari).

#### 3.4.2 Subtheme—Increased positive outlook—found meaning and purpose

It appears that the sense of hopelessness, previously described by three participants, had disappeared. A sense of meaning and purpose was found, along with increased positivity. Hopelessness has been shown to be a risk factor for suicidal ideation, which is one of the symptoms needed for a diagnosis of depression according to the DSM-V (79, 80).

Therefore, by eliminating the sense of hopelessness and finding a sense of meaning, the risk of suicidal ideation may be reduced, which in turn reduces the number of diagnostic symptoms present (46).

Participants shared that they felt “a lot more positive and cheerful overall” (Jessica) and that it has given them “more of a positive outlook and helps me focus on my objectives” (Mark). Anika’s account showed the renewed sense of hope:

*“I think my outlook on life is better on the grounds that I do not think I’m going to end up sort of in a wheelchair or whatever, so I think I do feel more energised and it’s made me feel more positive.”* (Anika).

These improvements suggest an increase in aspects of psychological well-being such as mental well-being and depressive symptoms. These improvements in depressive symptoms would be in keeping with the findings from Tillery et al. (81) who reported a reduction from moderately severe depressive symptoms to no symptoms in a depression case study of the ketogenic diet. These findings are also in keeping with the improvement in mental well-being found by Unwin et al. (82) and the decrease in depression found by Danan et al. (16) when following a low carbohydrate and ketogenic diet. The observed improvements also support the reports of antidepressant effects found in mice models following a ketogenic diet (15, 83).

#### 3.4.3 Subtheme—Improved sleep

Sleep also improved for many participants on this diet. Some participants were struggling in meetings and were constantly falling asleep during the day. This appeared to resolve the longer they were following the diet and may also have been a result of their now stable energy levels. The literature suggests that some people experience a reduction in sleep duration when following a ketogenic diet, for example they sleep 7 h now instead of nine, but their sleep quality stays the same or improves, in that they wake up refreshed and more energized than before, therefore reducing morning sleepiness (84–88). One participant shared their sleep improvements:

*“I’m sleeping better, I struggle with insomnia and have done for four years and I’m definitely sleeping better.”* (Amari).

It is important to note that for some people, sleep may get worse before it gets better, especially in the first few weeks of the KD as the body moves into a state of ketosis (29, 89). For those with depressive symptoms or other diagnosed psychiatric illnesses this requires close monitoring as sleep deprivation or untracked sleep alterations can increase the possibility of experiencing negative psychiatric symptoms such as mania, hypomania, and psychosis (90–93). Therefore, it is important to always work alongside an experienced clinician when deciding to implement ketogenic metabolic therapy with the goal of reducing psychiatric symptoms and improving overall mental health.

#### 3.4.4 Subtheme—Increased energy and concentration

Concentration and energy levels improved for some participants whereas prior to starting the program these levels had been low. One participant who had been a student and studying throughout the program stated that:

*“I feel like I can concentrate a lot better because before I could never study on a night, it would have to be during the day because by 7 or 8 o’clock at night I was just completely drained whereas now I’m quite happy to keep reading until 9 or 10 o’clock at night. I just feel like everything is, concentration levels are much better.”* (Sarah).

Four participants stated that they have more energy overall and that they “feel a lot more energised” to do day to day things like walk their dogs (Jessica) or go to yoga (Anika). One reason for this may be that energy levels when in ketosis and keto-adapted, remain stable throughout the day and do not rely on glucose to provide energy as there is a consistent supply of fat store derived ketones to use as energy (94). Campbell and Campbell (6) found that 25% of their participants experienced increased energy when following a ketogenic diet for their bipolar symptoms. Overall, this improvement in both energy and concentration is encouraging as both lack of energy and reduced concentration are symptoms that may be indicative of depression (46).

#### 3.4.5 Subtheme—Increased confidence and self esteem

Alongside these improvements, some participants’ confidence that was lost prior to the program, began to make a comeback and self-esteem improved also. Two participants from the depressive symptoms group mentioned that they felt better about themselves and that they feel more confident since following the program, “feeling better about myself is its own reward” (Philip). Jessica struggled prior to the program with low confidence, and since then she shared that:

*“When I left work my confidence was just rock bottom, I just though oh where is me gone? And now I just feel that me is coming back really.”* (Jessica).

These improvements may be related to the diet change and the effect of ketones, but they may also be attributed to a sense of achievement in meeting their weight loss goals and improving their physical and mental health. These improvements are in keeping with the findings of Protogerou et al. (95) in individuals following a zero-carbohydrate diet.

#### 3.4.6 Subtheme—Increased well-being and feeling in control of life

Increases in psychological well-being, a sense of calm, equilibrium, and patience were also observed among at least five participants which is in keeping with the earlier mentioned work by Harvey et al. (24). Experiencing calmness when in ketosis is not a new phenomenon and research suggests that this may be because ketones can reduce neuronal excitability (96). Perhaps this is what participants experienced when they mentioned a sense of calm, patience, and less frustration. This increased sense of calm and tranquility experienced by participants is the opposite of agitation which is a symptom that may be indicative of depression (46).

It is also possible that the routine associated with following the ketogenic diet gave participants a greater sense of control over their diet and their health. Perhaps they were able to form healthy habits as once established, routines and habits require little effort to maintain (97). This may have contributed to increased patience with others as they were less worried and experienced less frustration and decision fatigue when it came to self-care and diet choices, leading them to exhibit a sense of increased well-being.

Whitney stated that she is “no longer in the same place as when I started, much happier” and Mark summed up his heightened well-being:

*“You know the song Park Life by Blur, where it says you should cut down on your pork pies mate get some exercise, and it talks about the birds and it giving him an enormous sense of well-being, and that always resonates with me in my head, it should be called “pork life” not park life, the enormous sense of well-being that you get.”* (Mark).

Overall, improvements in psychological well-being were observed by many participants from both the healthy adults group and those with depressive symptoms. Improvements were noted in aspects of psychological well-being that were low prior to the program start, for example self-esteem, motivation, confidence, and a sense of meaning and purpose. The improvements experienced here are in keeping with the findings stated earlier from Harvey et al. (24), Newson and Parody (27), and Wong et al. (28).

These psychological improvements may have been a result of the dietary changes and ketone effects on a biological level. However, on a psychosocial level, improvements may also have been a consequence of achieving their weight loss goal, taking control of their health by following a diet, or contributing their data as part of a wider research study.

### 3.5 Theme 4—It becomes a lifestyle

This theme discusses how the diet becomes a lifestyle over time for participants. There were some initial implementation difficulties that will be discussed later, but overall, it appears that participants were able to easily follow the diet once they understood how to apply and integrate it into their life. On average it takes 66 days or 9 weeks to create an automatic habit, which suggests that for those who continued the study for the duration of the intervention (12 weeks), they may have created a new habit, of following the diet (97). Perhaps this is what helped them to turn it into a lifestyle. These findings are in keeping with the qualitative findings of Newson and Parody (27) who looked at the experience of a LCD in those with T2D. Their participants stated that the diet was difficult initially but then it became sustainable. Participants also noted that they no longer craved carbohydrates and looked at a LCD as a lifestyle.

#### 3.5.1 Subtheme—Educated participants and easy program to follow

Participants shared that the diet wasn’t that difficult “once you get the hang of it” (Diane), and that “it does not seem like particularly hard work” (Sarah). Over time it became a lot easier to understand what participants could and could not eat and it therefore became a lot more “instinctive” (Anika). Mark stated that:

*“This is so easy to do, it’s a no brainer and I do not know why it is not out there.”* (Mark).

This is in keeping with the works of Wong et al. (28) in comparison to other diets that they had tried in the past, the KD was easier to follow, tastier, and overall, was more enjoyable.

However, in order for it to become a lifestyle, education about the diet, how it works and how to implement it was crucial. Initially, participants followed education videos provided in the program, learnt to read product labels and understand them, and calculated carbohydrates, calories and macronutrients using a notebook or an online tracker app such as MyFitnessPal or Cronometer. Over time, and with practice, the need to do this repeatedly reduced. This may be because participants had learnt the macronutrient composition of most of their foods and therefore only needed to do this when eating something that they would not usually eat, such as when out at restaurants or on holidays. Cadario et al. (98) found that in the general population, individuals tend to eat the same breakfast every day while seeking more variety for other meals. This lends to the idea that once participants found one or two suitable breakfast options, the frequency of tracking may have dropped. It is not certain, but perhaps this also happened for other meals in the day.

#### 3.5.2 Subtheme—Planning ahead—staying organized

Prior to the program start, some participants mentioned that they had no meal structure or food plan and that they often ate what was in front of them at work and therefore would later skip meals. After some time following the program, many participants found that the key to staying on track was to plan ahead and stay organized, and in some cases, cook or prepare food at home ahead of time such as making their own protein bars without sugar. Planning ahead and preparing food at home is not specific to the ketogenic diet however, as research shows that these actions are important for any dietary program to be successful (99). Meal planning and preparing food ahead of time is associated with a healthier diet overall (43).

Harriet shared that checking the menu and knowing where you will or can eat when out and about is a good way to keep this diet easy. Sarah stated that:

*“Even going into town, having an afternoon, you cannot have your cake and your coffee, you have just got to think ahead of what you are going to eat.”* (Sarah).

Eating out in restaurants and cafes was therefore no longer difficult or confusing. Participants were able to find meals on the menu that fit the ketogenic diet, or they would swap carbohydrate filled sides for leafy greens. Simple side swaps, switching a beer for a vodka and diet soda, skipping the bread, or leaving the chips behind meant that participants could still eat out and spend time with others, “you can order the food, just do not eat all of the carbs that come with it” (Diane). These findings are in keeping with the works of Wong et al. (28) who found that participants appeared to overcome these challenges over time by adjusting their routine.

#### 3.5.3 Subtheme—Making choices and being flexible

But for some, flexibility was key and ultimately the choices lay with the participants. Perhaps mastering this flexibility helped to keep them on track long term. These results replicate the findings from Newson and Parody (27) whose participants also mentioned that they allowed themselves some flexibility from time to time. Having said this, this flexible approach may not be possible for everyone using a ketogenic diet or ketogenic metabolic therapy to improve their mental health, psychological well-being or certain physical health conditions. More research is necessary in this area.

Participants were able to overcome implementation difficulties and navigate social situations in order to maintain the improvements in their physical and psychological health. Initially they may have been motivated by physical appearance changes but over the long term it appears it was other physical or psychological changes that kept them on track. Participants felt the diet was worth it given the health improvements they experienced.

*“If I’m left to my own devices, I’m absolutely fine.”* (Philip).

Having a greater “Why” for following the diet after the end of the program is key. Overall, this subtheme suggests that without the pressure of others, participants were able to make personal choices and decisions in line with their health goals.

### 3.6 Theme 5—Implementation difficulties

This theme identifies and discusses three main areas where difficulties were encountered when implementing the diet. As with any new diet or way or eating, a transition period is expected with some short-term obstacles to overcome. Learning about the macronutrients of foods, what and when to eat, all of this requires time. The ketogenic diet is no different. It takes time to learn about carbohydrates and the levels of these in each food and how they affect one’s individual blood glucose and ketone levels. In a study by Campbell and Campbell (6) looking at the implementation of ketosis in those with bipolar disorder, 22% of participants mentioned they encountered an adaption period before they experienced any positive effects from the diet.

#### 3.6.1 Subtheme—Diet implementation

The first challenge area was initiating the diet and getting into a state of nutritional ketosis. Participants found that it took some time before they felt they knew what they were doing. This is in keeping with the works of Campbell and Campbell (6) who found that 10% of their participants had difficulties implementing the diet initially.

In the first week to 2 weeks of implementing the ketogenic diet, there is a transition into ketosis which can give rise to some negative symptoms. This is better known as “keto induction” or experiencing the “keto flu” although it bears no similarity to the viral flu. These negative symptoms are transient and do not last long with resolution of symptoms reported from day 3 up to 4 weeks (29). From the data, as expected, some participants experienced these negative symptoms. These short-term symptoms included increased hunger in the first few days, wanting to urinate more than usual, loose bowels, and reduced energy to carry out day to day tasks. These symptoms “did not last very long at all” (Jessica), and “went away on their own” (Harriet). This is in keeping with findings from the literature which states that symptoms are less severe and do not last as long as expected (28, 95).

#### 3.6.2 Subtheme—Out and about

After short term symptoms subsided and participants entered a state of ketosis, the challenge participants then faced was how to fit the diet into their current lifestyle and how to overcome obstacles along the way. The second challenge area was trying to avoid carbohydrates day to day while out and about. Avoiding carbohydrates at social events was a challenge, especially as some felt they were missing out in some instances such as when others are eating dessert at a social lunch. Some participants simply did not want to come across as rude to their work colleagues. These situations eventually become easier once the lifestyle is implemented but initially it can be difficult. Many participants felt that restaurant and café meal options were predominantly carbohydrate based, which made it difficult initially to navigate the menu and choose ketogenic friendly options to enjoy. Participants mentioned that at the start of the diet, “it’s not great trying to keep the carbs low” (Diane) when out, and that if you stop anywhere, “most of the things available are sandwiches and sort of carb-based foods” (Anika). Diane said that:

*“The other options are there, it’s just, everything comes with piles of carbs quite honestly.”* (Diane).

This challenge also extended to holidays abroad where access to usual foods was restricted. Being “away from home was difficult” (Sarah) in the early days of the diet, for example, one participant stated that although restaurants had menus, “they are very limited” (Jessica) and “hotel options are not always all you might hope for” (Diane). However, once participants learnt what to eat and began to plan ahead, these challenges resolved.

#### 3.6.3 Subtheme—Diet dogma, societal impact and opinions of others

The third and final challenge area was overcoming societal norms and diet dogma, and this extended to the opinions of others like friends, family, and work colleagues. Though this is similar to Theme 4 - subtheme 3 “making choices and being flexible,” this subtheme relates to the impact of society and the outward world on the individuals’ food choices and decisions.

Following a diet that encourages the consumption of some foods that have been vilified in society (such as eggs, butter, bacon, and red meat) is difficult for some participants (100). This is understandable as mainstream nutritional advice has often been confusing and conflicting for people (101) which is not helped by the lack of adequate nutritional training for doctors to educate their patients (102). The idea of eating fat and reducing the amount of fruit in the diet was “really odd” and a “tricky thing to get your head around” (Amari). Diane had the same experience when it came to eating eggs:

*“I’m not sure, I was concerned about the wisdom about eating quite so many eggs.”* (Diane).

The pre-held beliefs about what foods to eat and not eat, as well as when to eat, extended to participants’ friends, family, and colleagues too. Once the participants were able to implement the diet it became the opinions of those around them that became the challenge. One participant mentioned that they felt pressured to eat the food given to them by a friend and “it was like, back to square one” (Harriet) with regards to hunger and cravings. Mark experienced this when out with friends:

*“They’ll ask me why aren’t you eating and I’ll say well I’m not hungry and then I do not know if we will end up going down a rabbit hole.”* (Mark).

Philip also noted that he had no issue when on his own but that:

*“If there are other people around or if I go and see a friend or something like that, I find I’m having to say no to cheesy chips.”* (Philip).

Overall, participants were able to navigate and overcome these three main challenges when implementing the diet.

Food and eating for many in society is a social occasion in the presence of other people. Eating with family in the evenings or eating with friends at the weekends has been shown to facilitate social bonding and increases satisfaction with life (103). It has been reported that individuals are influenced by what and how much those around them eat (104). For example, there is research to suggest that eating with a partner who chooses ‘unhealthy’ foods, may negatively influence an individual’s decision to eat ‘healthy’ foods (105) and that societal norms can also have a negative impact on an individual’s food choice and intake (106).

A study by Vue et al. (107) found eight ‘need states’ in which individuals eat which range from a basic need for food, to social expression, celebration or to gain recognition suggesting that individuals eat for both physical and emotional or social needs. This suggests that there are other lifestyle obstacles and motivations, outside of just food as fuel, that get in the way of following a ‘healthy’ diet and can derail even the most focused individuals. Attempting to follow a diet that is different to those around you can be difficult, especially during the implementation phase.

## 4 Discussion

There is a growing body of evidence in support of using ketogenic metabolic therapy, and the KD as an adjunct to standard treatment for those with varying psychiatric illnesses. Studies looking at the effects of the KD on psychiatric conditions have been published as far back as the 1960’s (20) with more recent research focused on the diet’s effects on bipolar disorder, depression, schizophrenia and eating disorders (16, 18, 56, 108). The purpose of the current study was to review the accounts of participants who completed a KD intervention and to identify any common themes relating to their journey.

The complete array of biological mechanisms by which the KD works is not yet known and the conclusions from the literature are mixed, however, research suggests that KDs should be further tested as an intervention for some psychiatric conditions such as bipolar disorder and depression (7, 34). As there are a host of biochemical actions and reactions observed when in a ketogenic metabolic state, there is enough research to warrant a closer look at the possible effects on affect and other aspects of psychological well-being. A review by (109) of over 3,500 RCTs suggests that the effect that psychotherapy and pharmacotherapy have on psychiatric disorders is limited and that more research is necessary to identify other novel treatments for these conditions.

In the current literature there are very few qualitative studies that explore the accounts and lived experience of those following a low carbohydrate or ketogenic diet. Only one study looked specifically at healthy, non-obese, non-diabetic participants (24) and no studies were found that looked specifically at a depressed population following a low carbohydrate or ketogenic diet.

This current explorative qualitative study is the first to examine the accounts of following a KD in both healthy participants and those with depressive symptoms. The findings from this current study show that some improvements in mental health and psychological well-being were observed. Considering the thematic analysis was carried out with an essentialist realist epistemological stance, findings from this thematic analysis may be generalizable and repeatable if conducted with a similar methodology.

Through this current qualitative study, participants stated the strengths and limitations of following a KD to improve their health. The theme 1 subthemes, “low self-esteem, body satisfaction and self-worth,” and “low mood and hopelessness” were predominantly reported by those in the depressive symptoms group. In addition to this, those who experienced the theme 3 subtheme of “increased confidence and self-esteem” at the end of the intervention were also all from the depressive symptoms group. This might suggest that those with depressive symptoms experienced improvements in these areas over the duration of the intervention. Although no qualitative studies have looked at this population in relation to the ketogenic diet, the findings are in keeping with the thematic analysis carried out by Newson and Parody (27) whose participants with T2D experienced increased confidence levels.

It is interesting to note that those who expressed the theme 2 subtheme of “food addiction – addictive behaviour,” were all females. In the binge eating literature, a study by Levallius et al. (110) looking at addictive-like behaviors across genders found that 42% of females reported binge eating compared to 21% of males. To support this, a mouse study by Wei et al. (111) reported that female mice are more likely to have an addictive phenotype for sugar compared to male mice. Hussenoeder et al. (112) carried out a survey (*N* = 1,474) exploring anxiety and food addiction across genders and found that episodes of anxiety increase food addiction in females but not males. This may be because eating sweet foods in excess has been shown to reduce the effects of stress in females and not males (113). Further to this, females are more likely to report emotional eating, or eating because of anxiety compared to males (114). Perhaps the females in this current study experienced higher levels of addiction-like behavior as a result of this phenotype or in order to reduce anxiety and stress. Further research is necessary to fully understand this finding.

Overall, the accounts and obstacles that these participants faced while implementing the KD are mostly consistent with the current literature and the first author’s experience supporting individuals implementing ketogenic metabolic therapy and following a KD to improve the mental health. These accounts appear to cover most, if not all, of the challenges that are to be expected when starting a KD and implementing a lifestyle change like this.

## 5 Limitations and contribution to research

From the participants’ accounts in this study, it appears that the benefits and positive outcomes of this diet outweigh any negative side effects experienced. This is encouraging for those who are looking for adjunctive therapies to address and improve their depressive symptoms, or if they are simply looking to increase their overall physical and psychological well-being. However, further qualitative, and larger scale quantitative research is needed to develop a greater understanding of the challenges and obstacles that face individuals who start a ketogenic diet, for any health goal.

Further to this, understanding the individual tolerance and both the physical and mental response to the KD in the wider community, is warranted in order to design personalized approaches to dietary implementation. These tailored approaches should address, reduce, or eliminate both the personal and social challenges faced by so many when starting the KD, so as to make it easier to implement and maintain long term.

Overall, future ketogenic dietary protocols can be better informed from these results. Implementation difficulties at the commencement of the diet should be accounted for when designing new protocols. Individuals should be informed as to what to expect and support from peers, mentors or coaches should be available throughout the first few weeks at least until hunger and cravings reduce, individuals are comfortably in ketosis, and the timeframe for hypomania has passed. If individuals are using this dietary intervention to improve mental health symptoms, ongoing support by an experienced clinician is recommended.

In terms of risks for success and maintenance, the data from this study suggests that the implementation period was the most difficult to progress through. Therefore, alongside peer or mentor support, resources should be created to accompany future protocols. These resources could cover topics such as staying organized and planning ahead, eating out and about, how to manage social situations and how to overcome other lifestyle obstacles such as diet dogma.

Once participants made the KD a lifestyle, they presented as knowledgeable and educated on how to follow the diet but also were able to make personalized choices which made it easier to maintain.

From these results, it is clear that the KD can be beneficial for psychological well-being. As is known from the epilepsy and T2D research the KD can exhibit strong therapeutic effects for many illnesses. These results suggest that the KD can improve many aspects of psychological well-being. Importantly it would seem that in most individuals who might undertake it - not just those with moderate symptoms of depressive illness, but potentially those with low to no symptoms might experience enhanced well-being. This could be further explored with a broader range of well-being measures, and other mental health conditions such as ADHD, anxiety, and obsessive-compulsive disorder (OCD). Therefore, future promotion of the KD and ketogenic metabolic therapy should reflect this and be personalized to individuals who may benefit from its effects. Additionally, if the diet is presented in a personalized manner, tailored to the individual and targeting their specific symptoms, it may result in better adherence generally, but more specifically for those with poor mental well-being.

Overall, the results of this study will aid researchers to better understand how a low carbohydrate and ketogenic diet can be applied in people’s daily lives either by using an online program or with the support of mentors, coaches, and health professionals such as experienced clinicians, psychologists, dietitians, and nutritionists. The benefits, drawbacks, and changes to aspects of psychological well-being that may be experienced by participants, which have been indicated but relatively undetailed throughout the literature and the quantitative arm of this work are now better understood.

## Reflexivity statement

At the time of research project design, the researcher was working in acute inpatient psychiatric services as an assistant practitioner on both male and female wards. The researcher also took the position of ketogenic nutritional consultant in a private limited company from 2017 to 2023, the duration of this research project. Here, the researcher disseminated the current ketogenic and fasting literature into layman’s terms and educated the public with this information over a period of 6 years. The researcher also worked 1:1 and via groups with clients to initiate a ketogenic diet and fasting protocols, based on the scientific literature for the goals of fat loss and improved general health. The researcher has also personally followed a ketogenic diet since 2014. The initiation of this diet is what prompted this research project. In the final year of this research project, the researcher worked with clients who were implementing the diet with the goal of improving their mental health. Overall, this experience and these events may have aided the researcher in the design of this study and may naturally have shaped how the researcher developed codes and themes for the data in this study.

## Data availability statement

The datasets presented in this article are not readily available due to the qualitative nature of this research. Participants of this study did not agree for their full transcripts to be shared publicly. No requests for datasets are permitted.

## Ethics statement

The studies involving humans were approved by the University of East London, UREC 1718 87. The studies were conducted in accordance with the local legislation and institutional requirements. The participants provided their written informed consent to participate in this study.

## Author contributions

EB: Conceptualization, Formal analysis, Methodology, Writing – original draft. FH: Writing – review & editing. JW: Writing – review & editing. JB: Writing – review & editing. JT: Writing – review & editing.
